# Efficacy of combining intravitreal injections of ranibizumab with micropulse diode laser versus intravitreal injections of ranibizumab alone in diabetic macular edema (ReCaLL): a single center, randomised, controlled, non-inferiority clinical trial

**DOI:** 10.1186/s12886-020-01576-w

**Published:** 2020-07-29

**Authors:** Olga Furashova, Patrick Strassburger, Klio Ai Becker, Katrin Engelmann

**Affiliations:** 1 Department of Ophthalmology, Klinikum Chemnitz gGmbH, Flemmingstrasse 2, 09116 Chemnitz, Germany; 2 Hessemer MVZ, Augenmedizin Darmstadt, Martinspfad 72, 64285 Darmstadt, Germany

**Keywords:** Diabetic macular edema, Ranibizumab, VEGF inhibitor, Intravitreal injection, Micropulse diode laser

## Abstract

**Background:**

To evaluate if a combination therapy with micropulse diode laser (MPL) shows non-inferiority on visual acuity (BCVA) within 12 months in comparison to standard therapy, i.e. intravitreal injection of ranibizumab alone.

**Setting:**

Institutional. Prospective randomized single-center trial**.**

**Methods:**

Patients with diabetic macular edema (DME) received three intravitreal injections of 0.5 mg ranibizumab during the upload phase and were then randomised 1:1 to receive either the same dosage of ranibizumab (0.5 mg) injections pro re nata alone (IVOM-Group; *n* = 9), or with two additional treatments with micropulse diode laser (IVOM+Laser-Group; *n* = 10). The primary endpoint was change in BCVA after 12 months. Secondary endpoints were change in central macular thickness and overall number of ranibizumab injections.

**Results:**

BCVA increased significantly in both groups (IVOM: + 5.86, *p* < 0.001; IVOM+Laser: + 9.30; p < 0.001) with corresponding decrease in central macular thickness (IVOM: − 105 μm, *p* < 0.01; IVOM+Laser: − 125 μm; p < 0.01). Patients with additional laser treatment had better visual improvement (group comparison *p* = 0.075) and needed fewer ranibizumab injections (cumulative proportion of injections 9.68 versus 7.46 in IVOM-Group and IVOM+Laser-Group, respectively).

**Conclusion:**

Non-inferiority of combination therapy in comparison to standard therapy alone could be demonstrated. Patients with additional laser therapy needed fewer ranibizumab injections.

**Trial registration:**

Registered 10 February 2014 on ClinicalTrials.gov; NCT02059772.

## Brief summary

Combined treatment of diabetic macular edema with ranibizumab and micropulse diode laser is non inferior compared to standard therapy with ranibizumab alone. Patient with additional laser therapy needed fewer ranibizumab injections.

## Background

Diabetic retinopathy (DRP) and diabetic macular edema (DME) are diseases of the retina that are caused by complications of diabetes mellitus. DRP is the third most frequently reason for visual loss in industrialized countries [1, 2]. DME is caused by leaking macular capillaries and is the most common cause of visual loss in both proliferative and non-proliferative DRP.

Focal/grid macular laser photocoagulation is still sometimes used for therapy of vision-threatening DME [3, 4]. However, it does have considerable disadvantages because of its destructive nature [5].

In contrast, micropulse diode laser therapy (MPL) causes less damage to photoreceptors and retinal pigment epithelium cells [6]. Moreover, there is evidence for an additional positive effect of high wavelength (810 nm) of the diode laser supporting the recovery of cells especially those of the retinal pigment epithelium [7].

In 2011 the vascular endothelial growth factor (VEGF) inhibitor ranibizumab (Lucentis®, Novartis Pharma) was approved and recommended for the treatment of DME. Most of the patients treated with ranibizumab experience a qualitative as well as quantitative improvement of visual function within a few days after treatment already [8, 9]. However, the effect is only short-lasting thus requiring multiple follow-up injections, usually 7 to 9 in the first year of treatment [9, 10]. Moreover, up to now it remains open whether multiple injections may cause damage to photoreceptors and other parts of the retina [11, 12].

In several studies on patients with DME it was shown that the effect of MPL to that of the “standard” laser (gold standard) is at least comparable [13–16]. In clinical practice a two-time treatment with the diode laser is routinely performed in order to provide a long-lasting improvement of the visual function. Treatment with the VEGF-inhibitor ranibizumab leads to rapid improvement of DME. In contrast, the laser treatment is much slower and shows a weaker effect but the effect is more sustainable. Thus, the disadvantages of laser treatment might be circumvented by an initial combination therapy with ranibizumab.

In the ReCaLL study micropulse diode laser therapy was combined with the German Ophthalmological Society (DOG)-recommended first-line therapy, i.e. the intravitreal injection of the VEGF-inhibitor ranibizumab in dosage of 0.5 mg. The aim of the study was to investigate whether a combination therapy of micropulse diode laser treatment and intravitreal injection of the VEGF-inhibitor ranibizumab may improve the visual function more efficaciously than anti-VEGF injection alone and additionally minimize potential complications by reduction of injection frequency.

## Methods

ReCaLL (NCT02059772) was a single-center phase 4, prospective, open-label, randomized controlled study in Germany between April 2014 and December 2016. The study received approval from the independent ethics committee and was conducted in accordance with the Declaration of Helsinki and International Conference on Harmonization Good Clinical Practice guidelines. The study adheres to CONSORT guidelines. All participants provided written informed consent prior to inclusion in the study.

Patients included in this study met the following criteria: 1) diagnosed with non-ischemic DME; 2) BCVA between 35 and 89 on ETDRS charts or central retinal thickness > 300 μm as determined by spectral domain optical coherence tomography (SD-OCT). The exclusion criteria were: 1) severe ischemic maculopathy; 2) active neovascularization of iris or retina; 3) history of intravitreal injection of VEGF-inhibitor or steroids within the last 3 months; 4) pathologies of the anterior segment with reduced visual acuity (e.g. corneal opacification, advanced cataract); 5) other ocular pathologies with reduced visual acuity (e.g. central scars, age related macular degeneration, retinal vascular occlusion in medical history); 6) active or suspected ocular or periocular infections; 7) intraocular surgery or laser therapy within the preceding 6 months; 8) systemic steroid therapy within the last 3 months; 9) HbA1c greater than 10% or blood pressure above 170/110 mmHg.

After the first 3 monthly intravitreal injections of 0.5 mg ranibizumab (Lucentis®), subjects were randomised 1:1 to receive either standard treatment with ranibizumab pro re nata (IVOM-Group) or standard treatment plus two additional applications of micropulse diode laser (IVOM+Laser-Group) at Visits 5 and 6. Ranibizumab was injected in the same dosage of 0.5 mg in both groups. Visits were scheduled every 4 weeks until primary endpoint at 12 months. If retreatment criteria were met, follow-up injections of ranibizumab were given in both groups every 4 weeks until stability of BCVA was reached again. In IVOM+Laser-Group ranibizumab injections at Visits 5 and 6 were administered at least 30 min after laser photocoagulation. Physicians and technicians handling the patients were not masked about the grouping of the patients.

Table 1 demonstrates criteria for continuing or stopping ranibizumab treatment according to pro re nata (PRN). The primary endpoint was the mean change in BCVA over 12 months on ETDRS charts. Secondary endpoints were the mean change in CMT over 12 months determined by SD-OCT, and the overall number of treatments with ranibizumab.

**Table 1:** Re-treatment and stopping treatment criteria for pro re nata (PRN) regimen used in the study

Criteria for re-treatment	Criteria for stopping treatment
visual improvement	irreversible changes of central macula (i.e. atrophy, ischemia) without the perspective for visual improvement
decrease in CMT ≥10%^a^	BCVA < 35 as measured by ETDRS charts^b^
	BCVA of 100 on ETDRS charts
	lack of fluid in the area of the fovea

*CMT* central macular thickness; *BCVA* best corrected visual acuity; *ETDRS* Early Treatment Diabetic Retinopathy Study;

^a^ thickness evaluation under consideration of cysts and vitreoretinal membrane structures

^b^ with the exception of visual impairment due to other causes such as vitreous hemorrhage

**Fig. 1:**
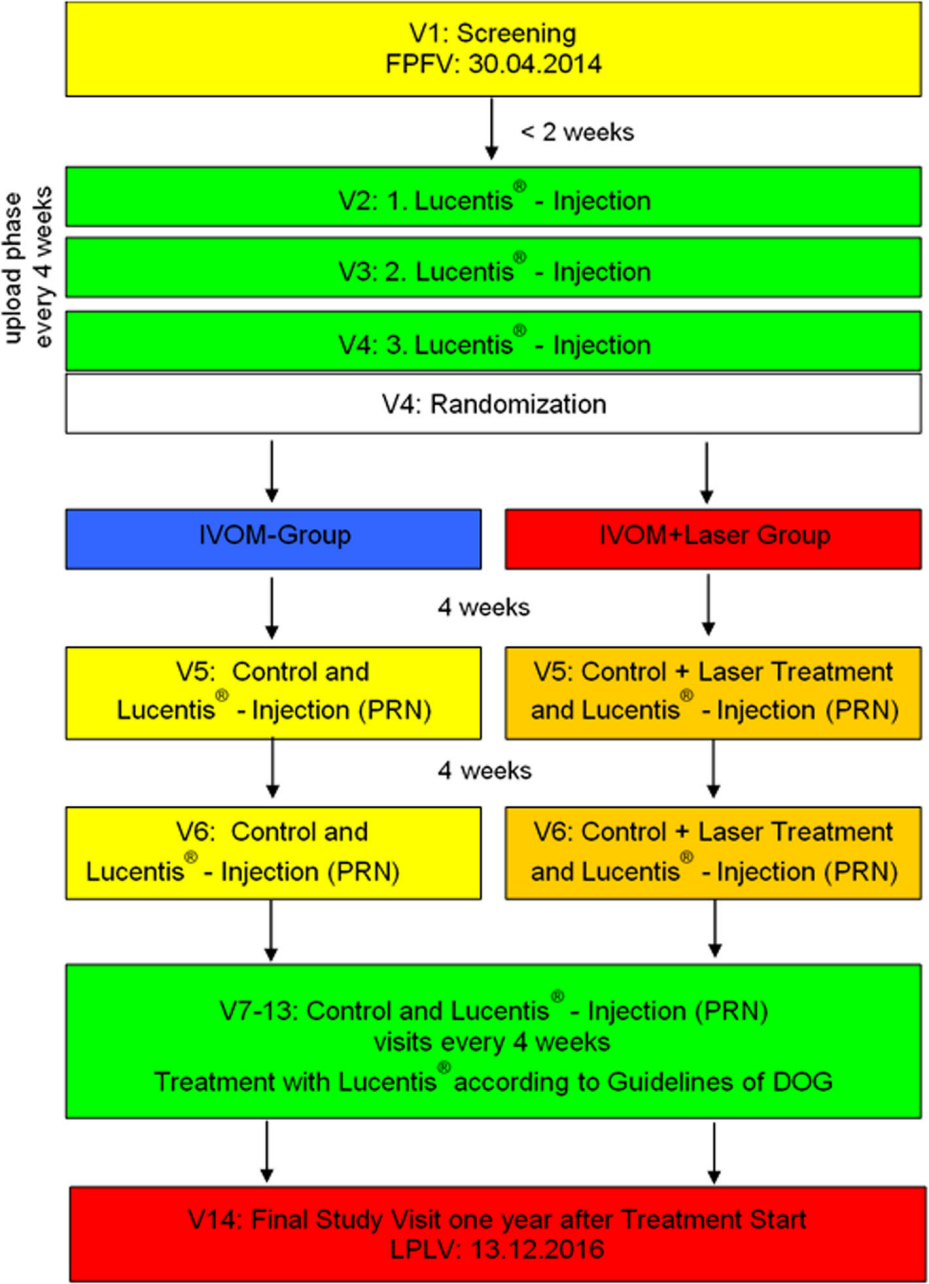
Trial flowchart. FPFV – first patient’s first visit; LPLV – last patient’s last visit; IVOM – intravitreal injection; PRN – pro re nata; DOG – Deutsche Ophthalmologische Gesellschaft (German Ophthalmology Society)

Figure 1 shows the trial flowchart with time frames of the present study.

### Laser treatment protocol

Laser treatment was performed with OcuLight SLx, Symphony Tri-Mode, IRIDEX Corp., Mountain View, CA, USA, at a wavelength of 810 nm using the magnifier Volk Optical Inc., Mentor, OH, USA. Treatment was performed as confluent laser photocoagulation of the macula without area of the fovea with a duty cycle of 15%, at doubled threshold energy and an exposure time of 200 msec.

### Statistical analysis

Approximately 50 individuals were to be enrolled in the study, i.e. 25 participants per study group. This sample size included a drop-out rate of 15% and had 90% power to detect a difference in means of 20%, assuming a standard deviation (SD) of 0.07 in a two-group t-test at 0.05 level of significance (one-sided). However, enrollment for this study was prematurely stopped after inclusion of 25 patients as the recruitment was substantially delayed. This was deemed to have no impact on the study outcome due to the exploratory character. All statistical analyses were performed on an explorative basis. Comparisons with no further statement of direction were tested at the 2-sided 5% significance level. Primary target analysis for non-inferiority was based on a one-sided 5% significance level referring to the stated direction in the statistical hypotheses. Analyses of continuous variables (BCVA, CMT) were performed by using Student’s t-test for independent variables (inter-group comparison of means or change of primary and secondary target variables) as well as Student’s t-test for dependent or related samples (intra-group comparison of mean at selected study visits). Visual acuity measurements were analyzed using ETDRS charts with final conversion to Snellen charts. IBM SPSS Statistics, version 23.0.0.0 for Windows (IBM, Armonk, NY, USA) was used to perform the analysis.

Efficacy endpoints were analyzed for the intent-to-treat set (ITT; all randomized participants) and for the per-protocol set (PPS; subjects who had complete data of primary and secondary target variables at the first and last visit, with no major protocol deviations thought to impact on the efficacy conclusions of the trial).

## Results

Overall, 17 of 25 included patients completed the study. For participants flow chart please see Table 2.

**Table 2:**
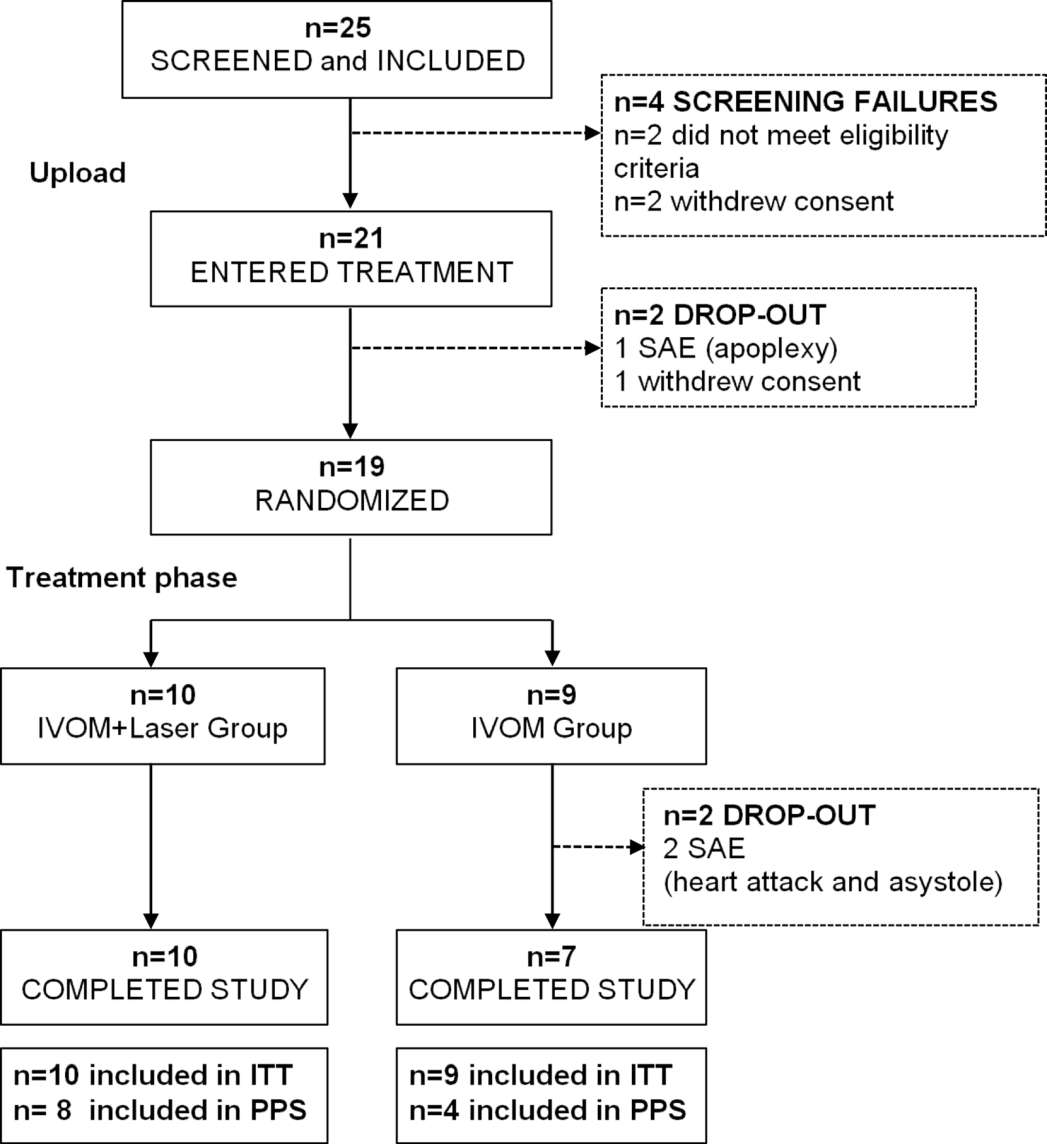
Participants flow chart

*ITT* intent-to-treat set (all randomized subjects); *PPS* per-protocol set (subjects with complete data of primary and secondary target variables at the first and last visit, with no major protocol deviations); *SAE* serious adverse event

Demographic and baseline characteristics of all randomized subjects were not statistically significantly different and are given in Table 3.

**Table 3:** Demographics and baseline characteristics (ITT, *N* = 19)

Variable		IVOM-Group (N = 9)	IVOM + Laser-Group(N = 10)	Total (N = 19)	***p-value***^*a*^
Age (years)	Mean ± SD	70.78 ± 8.96	70.70 ± 7.60	70.74 ± 8.03	*0.984*
Gender					
Male	N (%)	6 (66.7%)	8 (80.0%)	14 (73.7%)	
Female	N (%)	3 (33.3%)	2 (20.0%)	5 (26.3%)	
Race					
White	N (%)	9 (100%)	10 (100%)	19 (100%)	
Weight (kg)	Mean ± SD	92.83 ± 8.15	83.90 ± 12.92	88.13 ± 11.58	*0.093*
Height (cm)	Mean ± SD	173 ± 12	173 ± 8	173 ± 10	*0.860*
BMI (kg/m^2^)	Mean ± SD	31.13 ± 3.71	28.04 ± 2.38	29.51 ± 3.38	*0.043*
Diastolic blood pressure (mmHg)	Mean ± SD	82.13 ± 13.16	80.40 ± 9.38	81.17 ± 10.90	*0.750*
Systolic blood pressure (mmHg)	Mean ± SD	147.0 ± 14.22	151.1 ± 10.91	149.3 ± 12.28	*0.498*
HbA1c (%)	Mean ± SD	7.54 ± 1.51	6.80 ± 0.85	7.19 ± 1.26	*0.274*
Study eye					
Left	N (%)	7 (77.8%)	5 (50%)	12 (63.2%)	
Right	N (%)	2 (22.2%)	5 (50%)	7 (36.8%)	
BCVA	Mean	20/120	20/95	20/110	*0.478*
CMT (μm)	Mean ± SD	485 ± 170	434 ± 118	458 ± 143	*0.456*

*ITT* intent-to-treat set (all randomized subjects); *BMI* body mass index; *BCVA* best corrected visual acuity; *CMT* central macular thickness;

^a^comparisons between groups were done using Student’s t-test for independent variables

Tables 4 and 5 show the change in BCVA and CMT over the whole study period in both groups.

**Table 4:** Intra-group and inter-group comparison of BCVA during the study period

Dataset		Change in BCVA (mean ± SD)		
		Visit 1 → Visit 5	Visit 5 → Visit 14	Visit 1 → Visit 14
ITT	IVOM	4.11 ± 6.31 *(p = 0.087)*	2.86 ± 5.08 *(p = 0.187)*	5.86 ± 1.86 *(p < 0.001)*
	IVOM+Laser	4.30 ± 6.96 *(p = 0.083)*	5.00 ± 4.74 *(p = 0.009)*	9.30 ± 5.12 *(p < 0.001)*
	IVOM vs. IVOM+Laser	***p = 0.952***	***p = 0.387***	***p = 0.075***
PPS	IVOM	5.00 ± 6.88 *(p = 0.242)*	0.25 ± 5.12 *(p = 0.928)*	5.25 ± 2.06 *(p = 0.015)*
	IVOM+Laser	4.00 ± 7.29 *(p = 0.165)*	5.50 ± 5.21 *(p = 0.020)*	9.50 ± 5.26 *(p = 0.001)*
	IVOM vs. IVOM+Laser	***p = 0.824***	***p = 0.129***	***p = 0.158***

*ITT* intent-to-treat set (all randomized subjects); *PPS* per-protocol set (subjects with complete data of primary and secondary target variables at the first and last visit, with no major protocol deviations); *BCVA* best corrected visual acuity;

Comparisons between groups were done using Student’s t-test for dependent samples. BCVA changes are expressed in ETDRS charts for better statistical analysis

**Table 5:** Intra-group and inter-group comparison of CMT during the treatment period

Data set		Change in CMT (μm; mean ± SD)		
		Visit 1 → Visit 5	Visit 5 → Visit 14	Visit 1 → Visit 14
ITT	IVOM	−90.33 ± 114.65 *(p = 0.046)*	20.43 ± 79.61 *(p = 0.523)*	− 104.86 ± 68.76 *(p = 0.007)*
	IVOM+Laser	− 107.80 ± 56.24 *(p < 0.001)*	−16.70 ± 62.96 *(p = 0.423)*	− 124.50 ± 81.08 *(p = 0.001)*
	IVOM vs. IVOM+Laser	***p = 0.673***	***p = 0.299***	***p = 0.609***
PPS	IVOM	− 94.25 ± 77.47 *(p = 0.093)*	29.00 ± 34.28 *(p = 0.189)*	−65.25 ± 67.57 *(p = 0.149)*
	IVOM+aser	−99.13 ± 50.55 *(p = 0.001)*	−18.25 ± 71.21 *(p = 0.492)*	− 117.38 ± 82.71 *(p = 0.005)*
	IVOM vs. IVOM+Laser	***p = 0.897***	***p = 0.245***	***p = 0.304***

*ITT* intent-to-treat set (all randomized subjects); *PPS* per-protocol set (subjects with complete data of primary and secondary target variables at the first and last visit, with no major protocol deviations); *CMT* central macular thickness;

Comparisons between groups were done using Student’s t-test for dependent samples.

### BCVA

**Fig. 2:**
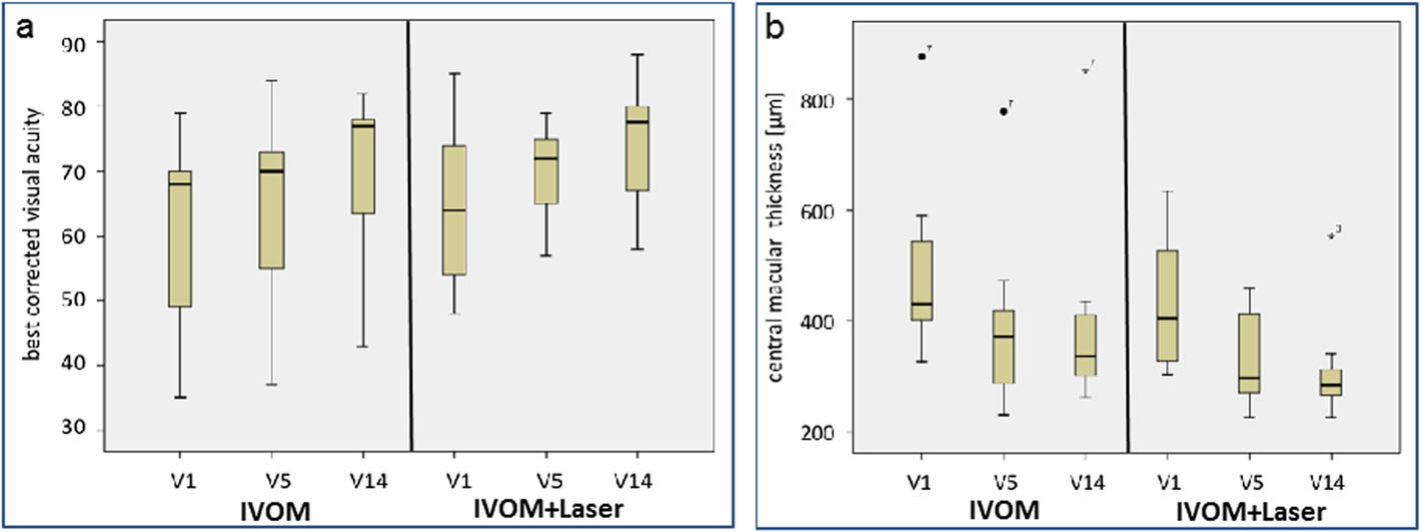
**a** - Boxplot for BCVA (best corrected visual acuity) measured with ETDRS charts (ITT, *n* = 19); no statistically significant difference could be observed between the groups at baseline (V1), after the upload phase (V5) and at the end of treatment (V14); BCVA values expressed in ETDRS charts; **b** - Boxplot for CMT (central macular thickness; μm; ITT, n = 19); no statistically significant difference could be observed between the groups at baseline (V1), after the upload phase (V5) and at the end of treatment (V14)

BCVA increased statistically significant in both treatment groups till month 12. No significant differences could be found in BCVA change between the two groups at any time (Fig. 2). Notably, a tendency was observed for a greater improvement in IVOM+Laser-Group than in IVOM-Group between baseline and end of treatment when considering the ITT (*p*-value for comparison of both groups at end of treatment 0.075), see Table 4.

### CMT

CMT decreased significantly in each treatment group over the study course. The effect of the treatment on CMT seems to be more prominent within the upload phase than later on and laser treatment seems not to contribute to this effect. Inter-group comparison of mean absolute CMT values as well as changes in CMT revealed no statistically significant difference between the groups (Fig. 2).

### Number of intravitreal injections

**Fig. 3:**
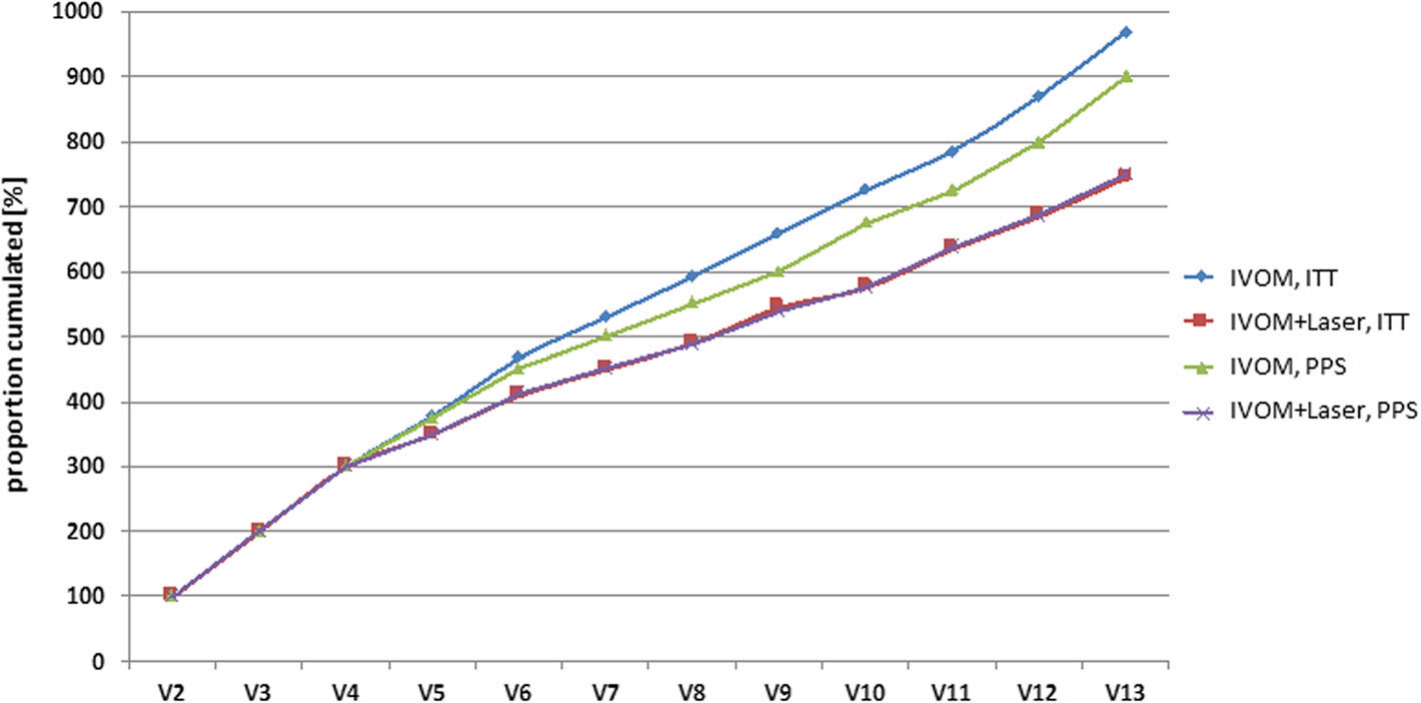
Cumulative proportion (%) of ranibizumab injections per visit. ITT - intent-to-treat set (all randomized subjects); PPS - per-protocol set (subjects with complete data of primary and secondary target variables at the first and last visit, with no major protocol deviations)

Subjects in IVOM+Laser-Group received less injections of ranibizumab than subjects in IVOM-Group (PPS: on average 9 injections per patient in IVOM-Group and 7.5 in IVOM+Laser-Group over a period of 12 months), see Fig. 3.

### Safety analysis

There have been 3 cases of corneal erosion and 1 case of conjunctival inflammation after ranibizumab injection. No ocular adverse events have been observed after laser treatment.

During the course of the study, three serious adverse events were documented. Two of them (apoplexy and myocardial infarction) were assessed as related to the study medication ranibizumab and for another one (asystole) relationship to the study medication could not be ruled out.

## Discussion

The present study showed non-inferiority of combined micropulse diode laser treatment with intravitreal injections of ranibizumab compared to ranibizumab injections alone in patients with DME.

Micropulse diode laser (MPL) has been shown to be more effective in improving morphology and function in patients with DME compared to conventional laser treatment [17]. Furthermore, MPL is known as a tissue-sparing laser treatment modality without laser-induced retinal damage. Chhablani et al. found significantly better retinal sensitivity parameters after subthreshold micropulse laser treatment compared to convetional continuous laser wave in DME patients [18]. A recent work of Midena et al. demonstrated also an additional anti-inflammatory effect of MDL [19].

Inagaki et al. showed recently, that combining anti-VEGF injections and minimally invasive laser treatment in DME results in good anatomical and functional improvement at 12 months [20]. While the visual gain in the study of Inagaki et al. was slightly worse (5.9 ETDRS letters), than in previous prospective studies for anti-VEGF therapy alone (6.6–10.3 EDTRS letters in REVEAl, RESTORE and RESOLVE), the mean number of injections was as low as 3.6 12 months after treatment.

In our study, we also observed a statistically significant improvement in BCVA after 12 months with a mean visual gain of 5.25 ETDRS letters in the IVOM-Group and 9.50 ETDRS letters in the IVOM+Laser-Group. Interestingly, the change in BCVA between the end of upload phase (Visit 5) and the end of treatment did not reach significance for IVOM-Group but for IVOM+Laser-Group indicating that the laser treatment exhibits an additional effect (Table 4).

Liegl et al. observed a similar BCVA improvement in DME patients treated either with ranibizumab alone or in combination with navigated laser photocoagulation (8.41 vs. 6.31 ETDRS letters, *p* = 0.258) [21]. However, in the group of combined treatment with ranibizumab injections and navigated laser photocoagulation, there were significantly less anti-VEGF injections required during the 12 months of follow up time (3.9 injections in the combination group vs. 6.9 in the injection group).

A recent retrospective study of Moiseiev et al. could also show a significant reduction in the burden of anti-VEGF injections when combining with MPL for DME treatment [22]. Kanar et al. demonstrated in a randomized clinical trial, that combining intravitreal aflibercept injections with subthreshold micropulse yellow laser for DME treatment results in fewer intravitreal injections and similar anatomic and functional outcome [23].

The results of our study confirm the findings of previous studies, showing fewer intravitreal injections of ranibizumab needed in the IVOM+Laser-Group compared to IVOM-Group (7.5 vs. 9.0, respectively). The mean number of intravitreal ranibizumab injections in our study was higher than in the cohorts of Liegl et al. and Inagaki et al. This might be explained by different PRN re-treatment criteria, timing of laser therapy as well as different patients’ characteristics including diabetes status and other systemic conditions (e.g. arterial hypertension), influencing the course of DME.

Nowadays, follow-up visits for injection treatments take place every 4 weeks. Furthermore, according to German regulations, patients are obliged to visit their ophthalmologist 1–3 days after every injection, thus making at least 2 visits pro injection necessary. This requires enormous efforts by patients, ophthalmic surgeons and ophthalmologists.

The results of our study support previous findings showing non-inferiority of combined treatment of DME with anti-VEGF injections and micropulse diode laser versus anti-VEGF injections alone. We could not find any differences in anatomical and functional outcome in both groups after 1 year of treatment. Furthermore, additional micropulse tissue-saving laser treatment in DME seems to reduce the number of intravitreal injections, thus improving the quality of life of both ophthalmologists and their patients as well as reducing the economic burden.

It should be mentioned, that in IVOM+Laser-Group, the patients required a mean of 1.5 fewer injections but had two additional laser treatment visits. However, regarding the known risks of possible ocular and systemic complications of both treatment modalities as well as the costs of laser treatment compared to intravitreal injections, we believe, that combined therapy still has its economic benefits. Furthermore, our study represented the disease treatment course only over 12 months. It would be interesting to know, whether the patients in the IVOM+Laser-Group had further benefit of fewer injections after 12 months.

One major limitation of this Phase 4 study was the low sample size. This might have compromised the study results and their validation. It is well known, that the treatment outcome in DME is associated with baseline characteristics such as disease duration, HbA1c, blood pressure level, BCVA, CMT, etc. In our study, both treatment groups showed no statistically significant differences in the baseline parameters, but this might also have been compromised by the small sample size.

The study was planned to include 50 patients but finally only 19 were randomized in total and even only 12 could be included in the PPS group. High efforts were required to enroll an appropriate number of eligible and willing patients for the study in a reasonable timeframe. This was accompanied by lack of compliance by some of the study participants. But in the end, this mirrors the conditions of the patient population, i.e. elderly people with diabetes and a series of co-morbidities for whom it would be of great benefit to reduce the strenuous effort caused by frequent visits to the ophthalmologist and repeated treatments.

## Conclusion

Our results support the findings of other study groups, who also observed a benefit of additional micropulse diode laser treatment in DME patients treated with ranibizumab. While the functional and anatomical results show non-inferiority of combined treatment versus anti-VEGF injections alone, the injection frequency can be reduced suggesting better quality of life and economic benefit.

## Data Availability

The datasets during and/or analysed during the current study available from the corresponding author on reasonable request.

## Abbreviations

BCVA: Best Corrected Visual Acuity
DME: Diabetic Macular Edema
IVOM: Intravitreal Injection
VEGF: Vascular Endothelial Growth Factor
DRP: Diabetic Retinopathy
MPL: Micropulse Laser
DOG: Deutsche Ophthalmologische Gesellschaft = German Ophthalmological Society
ETDRS: Early Treatment Diabetic Retinopathy Study
SD-OCT: Spectral Domain Optical Coherence Tomography
PRN: pro re nata
CMT: Central Macular Thickness
ITT: Intent-To-Treat Set (all randomized subjects)
PPS: Per-Protocol Set (subjects with complete data of primary and secondary target variables at the first and last visit, with no major protocol deviations)
SAE: Serious Adverse Event
SD: Standard Deviation
